# Modulation of Platelet Activation and Thrombus Formation Using a Pan-PI3K Inhibitor S14161

**DOI:** 10.1371/journal.pone.0102394

**Published:** 2014-08-12

**Authors:** Wenxiu Yi, Qiang Li, Jian Shen, Lijie Ren, Xiaohui Liu, Qi Wang, Sudan He, Qingyu Wu, Hu Hu, Xinliang Mao, Li Zhu

**Affiliations:** 1 Cyrus Tang Hematology Center, Collaborative Innovation Center of Hematology, MOH Key Lab of Thrombosis and Hemostasis, Jiangsu Institute of Hematology, the First Affiliated Hospital, Soochow University, Suzhou, China; 2 Department of Pathology and Pathophysiology, Zhejiang University, Hangzhou, China; University of Kentucky, United States of America

## Abstract

The phosphatidylinositol 3–kinase (PI3K) signaling pathway is critical in modulating platelet functions. In the present study, we evaluated the effect of S14161, a recently identified pan-class I PI3K inhibitor, on platelet activation and thrombus formation. Results showed that S14161 inhibited human platelet aggregation induced by collagen, thrombin, U46619, and ADP in a dose-dependent manner. Flow cytometric studies showed that S14161 inhibited convulxin- or thrombin-induced P-selectin expression and fibrinogen binding of single platelet. S14161 also inhibited platelet spreading on fibrinogen and clot retraction, processes mediated by outside-in signaling. Using a microfluidic chamber we demonstrated that S14161 decreased platelet adhesion on collagen-coated surface by about 80%. Western blot showed that S14161 inhibited phosphorylation of Akt at both Ser473 and Thr308 sites, and GSK3β at Ser9 in response to collagen, thrombin, or U46619. Comparable studies showed that S14161 has a higher potential bioavailability than LY294002, a prototypical inhibitor of pan-class I PI3K. Finally, the effects of S14161 on thrombus formation *in vivo* were measured using a ferric chloride-induced carotid artery injury model in mice. The intraperitoneal injection of S14161 (2 mg/kg) to male C57BL/6 mice significantly extended the first occlusion time (5.05±0.99 min, n = 9) compared to the vehicle controls (3.72±0.95 min, n = 8) (P<0.05), but did not prolong the bleeding time (P>0.05). Taken together, our data showed that S14161 inhibits platelet activation and thrombus formation without significant bleeding tendency and toxicity, and considering its potential higher bioavailability, it may be developed as a novel therapeutic agent for the prevention of thrombotic disorders.

## Introduction

Platelets play a critical role in atherothrombosis that leads to myocardial infarction and ischemic stroke [1], [2]. Once vascular injury occurs, the binding of the platelet glycoprotein (GP)Ib complex to von Willebrand factor (VWF) on the injured vessel wall initiates platelet tethering and subsequent adhesion [3]. The exposed collagen in the vascular wall and locally generated thrombin activate platelets and initiate hemostasis. The binding of collagen to GPVI on platelets results in receptor clustering and thereby stimulates phosphorylation of specific tyrosine residues within an associated trans-membrane protein, the Fc receptor γ-chain (FcRγ-chain). This leads to the recruitment of signaling proteins such as Src kinase, the tyrosine kinase Syk, PLCγ2, phosphatidylinositol 3-kinase (PI3K) and mitogen activated protein kinases (MAPKs), resulting in the inside-out activation of the integrin αIIbβ3 and the release of the secondary mediators, such as ADP and thromboxane A2 (TxA2), culminating in platelet aggregation mediated by fibrinogen [4], [5], or other ligands binding to αIIbβ3 [6], [7]. The modulation of platelet activity using specific pharmacological agents has proven to be a successful strategy for the prevention of thrombosis. The successful introduction of antiplatelet drugs, such as antagonists of ADP and αIIbβ3, and inhibitors of COX-1 and phosphodiesterase, has led to considerable improvements in the management of cardiovascular diseases [8]. However, the risk of uncontrolled bleeding due to their inherent antihemostatic effects limited their clinical use [9]. Therefore, tremendous effort has been made in the past years on the identification of novel pharmacological reagents with both effective and safe antiplatelet effect.

The recent search for compounds to prevent platelet activation has been focusing on the ones that modulate PI3K pathway. PI3K is a critical transmitter of intracellular signaling during platelet activation [10]–[12], capable of triggering a wide variety of responses like phosphorylation of pleckstrin, activation of PLCγ [13], Rap1b and AKT [14]–[17], and mediating several important platelet responses like platelet shape change and stabilization of platelet aggregation [18]. Platelets contain PI3K class IA (p110α, p110β and p110δ), class IB (p110γ), and class II (C2α) [19]. Knock-out mouse models showed that PI3Kγ acts as an important effector of P2Y12 while PI3K-IA as a key effector of collagen receptors [10], [12]. PI3K activation leads to the phosphorylation of AKT or protein kinase B, which is a critical player in platelet function [20], [21]. Targeting the PI3K/AKT is thus becoming an emerging strategy in the control of platelet-associated diseases. So far, more than 50 inhibitors for the PI3K/AKT/mTOR pathway are under clinical evaluation at different phases [22]. However, none of these PI3K inhibitors has been approved for modulating platelet activation and thrombus formation clinically.

We recently identified S14161, or 8-ethoxy-2-(4-fluorophenyl)-3 -nitro-2H-chromene, as a novel PI3K inhibitor, that displays promising effects against multiple myeloma and leukemia cells with minimal toxicity [23]. However, whether S14161 affects platelet activation and thrombus formation is unknown. Therefore, in this study, we investigated the effect of S14161 on platelet activation in response to a variety of agonists and thrombus formation. We showed that S14161 significantly inhibited agonist-induced platelet activation and thrombus formation via inhibiting PI3K/AKT pathway.

## Methods and Materials

### Animals and human samples

All animal procedures were approved by the University Committee on Animal Care of Soochow University (20140431) and each researcher followed the approved procedures when caring for or sacrificing mice and when conducting protocols involving mice. C57BL/6 mice were housed at a constant room temperature, humidity, and light cycle (12∶12 hr light-dark) with free access to water and were fed chow diet. At age 6–8 weeks, mice were randomly grouped into S14161- or vehicle-treated groups. Anesthesia using 7% chloral hydrate was applied to avoid any pain of animals during procedures. After the experiments, the animals were euthanized by CO2 inhalation. Human venous blood was obtained from healthy donors in accordance with the Declaration of Helsinki and the permission from the University ethical committee of Soochow University. The written informed consent was obtained from all participants.

### Reagents

S14161 was synthesized and purified by high performance liquid chromatography (HPLC) as reported previously [24]. Collagen, thrombin, and ADP were purchased from Chrono-Log Corp (Havertown, PA, USA). U46619 was purchased from Calbiochem (La Jolla, CA, USA). Antibodies against AKT, phospho-AKT Thr308, phospho-AKT Ser473, phospho-GSK3β Ser9, were purchased from Cell Signaling Technologies (Danvers, MA). The pan-PI3K inhibitor LY294002 was from Beyotime (Nantong, China). TRITC-Phalloidin was from Sigma-Aldrich (St. Louis, MO, USA). PE-conjugated anti-human CD62P, FITC-conjugated polyclonal anti-human fibrinogen, and Annexin V-APC were purchased from BD Biosciences (San Jose, CA).

### Platelet isolation and aggregation

Human venous blood was obtained from healthy donors and anticoagulated 1∶5 with ACD (65 mM Na3 citrate, 70 mM citric acid, 100 mM dextrose, pH 4.4). Platelet-rich plasma (PRP) was obtained by centrifuging at 900 rpm for 20 minutes [25]. Gel-filtered platelets were prepared as described previously [26]. Briefly, PRP was applied to the column that was packed with Sepharose 2B beads in phosphate-buffer saline (PBS) in a column and. Platelets were eluted using Tyrode's buffer to a series of 1.5-mL tubes. The collected platelets in each tube were counted, combined, and adjusted to 2.5×10^8^/mL using Tyrode's buffer. Platelets aggregation was performed in a ChronoLog aggregometer (Havertown, PA). Platelets were preincubated with vehicle or S14161 for 10 min at 37°C in a cuvette. Before adding agonists, CaCl_2_ (1 mM) and fibrinogen (200 µg/ml) were added. Aggregation assay was started with 0% aggregation baseline and then an agonist was added to observe the percentage of platelet aggregation with stirring at 900 rpm.

### Platelet viability assay

Platelet viability assay was described as previously [27] and AlamarBlue cell viability reagent was used as described by the vendor's manual (Invitrogen, Carlsbad, CA, USA). Briefly, gel-filtered platelets (2.5×10^8^/ml) were preincubated with S14161 or vehicle and then incubated with AlamarBlue reagent for 4 hours at 37°C. Upon entering cells, resazurin, the major component of AlamarBlue reagent, is reduced to resorufin, a compound that is red in color and highly fluorescent. When platelets are alive they maintain a reducing environment within the cytosol and the generated fluorescence was acquired on a SpectraMax Microplate Reader (Molecular Devices, Sunnyvale, CA, USA). The excitation and emission wavelengths were 570 nm and 585 nm, respectively. Tyrode buffer was used as a background control.

### Phosphatidylserine (PS) Externalization Assay

PS Externalization Assay was performed as previously described [27]. Gel-filtered platelets (2×10^7^/ml) were preincubated with S14161 or dibucaine (500 µmol/ml) at room temperature for 15 min. Annexin V binding buffer (0.01 M HEPES, pH 7.4, 0.14 M NaCl, 2.5 mM CaCl_2_) was mixed with pre-treated platelets and Annexin V-APC at a 50∶50∶1 ratio. Samples were gently mixed and incubated at room temperature for 15 min in the dark before being analyzed on a flow cytometer (FACSCalibur^M^, Becton Dickinson, San Jose, CA, USA).

### Immunoblotting

Aliquots of gel-filtered platelets (250 µl, 2.5×10^8^/ml) were pre-incubated with vehicle or S14161 for 10 minutes and stimulated by agonists under stirring at 37°C. The reaction was stopped by adding RIPA buffer (1% Triton X-100, 1% deoxycholate, 0.1% SDS, 10 mM Tris, 150 mM NaCl containing protease inhibitors and phosphatase inhibitors). After heating to 100°C for 10 minutes, proteins were separated on 10% SDS polyacrylamide gel electrophoresis (SDS-PAGE) and transferred to a nitrocellulose membrane (Bio-Rad). The membrane was incubated with antibodies at 4°C overnight followed by a corresponding secondary antibody (goat anti-rabbit IRDye 800CW or goat anti-mouse IRDye 800CW). The densitometric band scanning was performed using Odyssey infrared imaging system (LI-COR Biosciences, Lincoln, NE, USA).

### Soluble fibrinogen binding and P-selectin expression

Gel-filtered human platelets were adjusted to 2×10^7^/ml by adding Tyrode's buffer, and were incubated with or without S14161 (10 µM) for 10 minutes at 37°C. The platelets were then stimulated with convulxin or thrombin in the presence of FITC-labeled fibrinogen or PE-labeled CD62P at room temperature for 15 minutes and immediately fixed with 4% paraformaldehyde. Platelet-bound fluorescence was analyzed using a BD FACS Calibur Flow Cytometer.

### Platelet spreading on immobilized fibrinogen

Glass coverslips were coated with 10 µg/ml fibrinogen in 0.1 M NaHCO_3_ (pH 8.3) by soaking at 4°C overnight, and then washed with PBS. After incubation with or without S14161 at 37°C for 30 minutes, 2×10^7^/ml platelets in Tyrode's buffer were allowed to spread on the fibrinogen coated coverslips for 60 minutes at 37°C. After washed five times with PBS, adherent platelets were fixed with 4% paraformaldehyde, and stained with TRITC-labeled phalloidin containing 0.1% Triton X-100 at room temperature for 2 hours. Coverslips were mounted on the slides, and platelet spreading was observed under a florescence microscope (Olympus, FSX100).

### Clot retraction

As previously described [28], platelet-rich plasma (3×10^8^ platelets/mL) obtained by centrifugation of whole blood at 900 rpm for 20 minutes was incubated with S14161 for 30 minutes at 37°C. Fibrinogen was then added to a final concentration of 2 mg/mL, and the PRP were dispensed in 0.25 mL aliquots into siliconized glass tubes. Clot retraction was initiated by 1 U/mL of thrombin and allowed to proceed at 37°C. Clot retraction was monitored at indicated time points using a digital camera. The extent of retraction was quantified using Image J software developed by National Health Institute (Bethesda, MD).

### Microfluidics chamber

The human whole blood was used for platelet adhesion assay under flow. The experiments were performed as described previously [27] using BioFlux 200 setup (Fluxion Biosciences, USA) following manufacture's instruction. Briefly, the channels were primed and coated with collagen I (200 µg/ml) for 1 hour at room temperature and then blocked with PBS containing 0.5% BSA for 1 hour. The human whole blood was treated with S14161 or vehicle and labeled with calcein-AM (Molecular Probes, Eugene, OR, USA) at a final concentration of 4 mM for 30 minutes at room temperature. Blood was perfused in the channels at 1000 s^−1^ and observed under a fluorescent microscope for platelet adhesion and aggregates.

### FeCl_3_-induced carotid artery injury model

FeCl_3_-induced carotid artery injury was performed as previously described with some modifications [27], [29], [30]. Briefly, S14161 (2 mg/kg) or vehicle was administered intraperitoneally to mice (C57BL/6, age 6–8 weeks), and after anesthesia using 7% chloral hydrate, the left carotid artery of mice was surgically exposed by blunt dissection. A 1.0×2.0-mm filter paper soaked in 7.5% FeCl_3_ was applied to the surface of the adventitia of the exposed artery for two minutes. After removal of the filter paper, the artery was washed with PBS and an imaging ultrasound gel (MS400-0090; VisualSonics) was placed in the surgical wound to allow Doppler monitoring. The artery was identified using a small animal blood flow transducer (MS400, 18–38 MHz; VisualSonics) and the color Doppler mode of the VisualSonics Vevo model 2100 flowmeter. Time to occlusion of the carotid artery after the application of 7.5% FeCl_3_ was measured using Visual Sonics View 2100. The operator was blinded to mice that infused either S14161 or vehicle while performing all experiments.

### Tail bleeding time in mice

Tail bleeding times were determined as previously described [27]. Briefly, S14161 or vehicle was administered intraperitoneally to mice (C57BL/6, age 6–8 weeks). Forty-five minutes later, mice were anesthetized with 7% chloral hydrate and placed prone on a heating pad from which the tail protruded. The distal 5 mm of the tail was transected and immediately immersed in 12 ml 0.9% sodium chloride for 10 min at 37°C, and the time to bleeding cessation was recorded.

### Statistical analysis

Data were analyzed by using GraphPad Prism 5.0 software and presented as means ± standard error of the mean. The statistical significance was determined by one-way ANOVA analysis of variance with the Bonferroni post test for multiple groups. The Student t test was used to calculate P values for differences. Differences were considered significant at P<0.05.

## Results

### S14161 inhibits platelet aggregation

**Figure 1:**
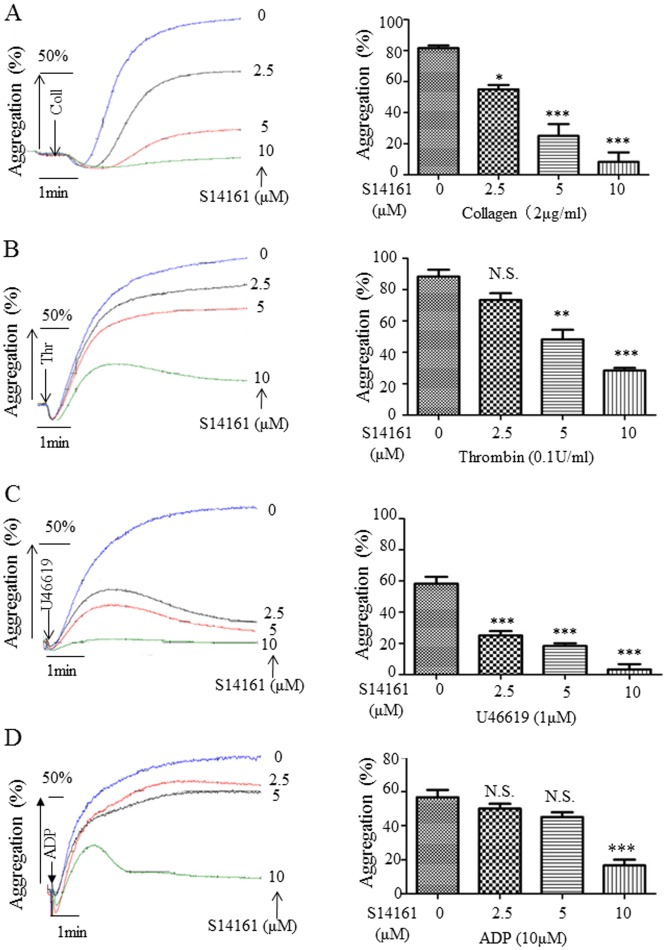
S14161 inhibited platelet aggregation. Gel-filtered human platelets (2.5×10^8^/mL) were preincubated for 10 minutes with different concentrations of S14161 (2.5 µM, 5 µM and 10 µM) or vehicle (DMSO). Platelet aggregation was initiated with collagen (2 µg/mL), thrombin (0.1 U/mL), U46619 (1 µM), or ADP (10 µM). The aggregation curves are the representatives of at least three individual experiments. Means ± standard errors of the mean of platelet aggregation percentage from three experiments are plotted in the bar charts; *P<0.05, **P<0.01 and ***P<0.001 as compared with control. N.S. means no significance (n = 3).

PI3K is critical in mediating platelet activation by a variety of agonists and targeting PI3K could be a promising strategy in the treatment of diseases in association of overactivated platelets and prothrombotic condition. Therefore, we asked whether S14161 is able to suppress platelet activation and thrombus formation. We first examined the potential effect of S14161 on platelet aggregation. Gel-filtered human platelets were incubated with S14161 (2.5, 5 and 10 µM) or solvent alone for 10 minutes at 37°C and then stimulated with collagen (2 µg/ml), ADP (10 µmol/L), U46619 (1 µmol/L), or thrombin (0.1 U/ml). As shown in Figure 1, platelet aggregation triggered by all these four agents was inhibited by S14161 in a dose-dependent manner. For example, the maximal aggregation induced by collagen were decreased by 32.7% (P<0.05), 59.4% (P<0.001) and 89.2% (P<0.001) when preincubated with 2.5, 5 and 10 µM of S14161, respectively (Fig. 1A). The IC50s of S14161 in inhibiting agonist-induced platelet aggregation were 3.79±1.17 (µM) for collagen (2 µg/ml), 6.32±0.51 (µM) for thrombin (0.1 U/ml), and 1.38±0.97 (µM) for U46619 (1 µM). S14161 also inhibited platelet aggregation induced by GPVI selective ligand convulxin and CRP, PAR1 agonist SFLLRN, and calcium ionophore A23187 (data not shown).

**Figure 2:**
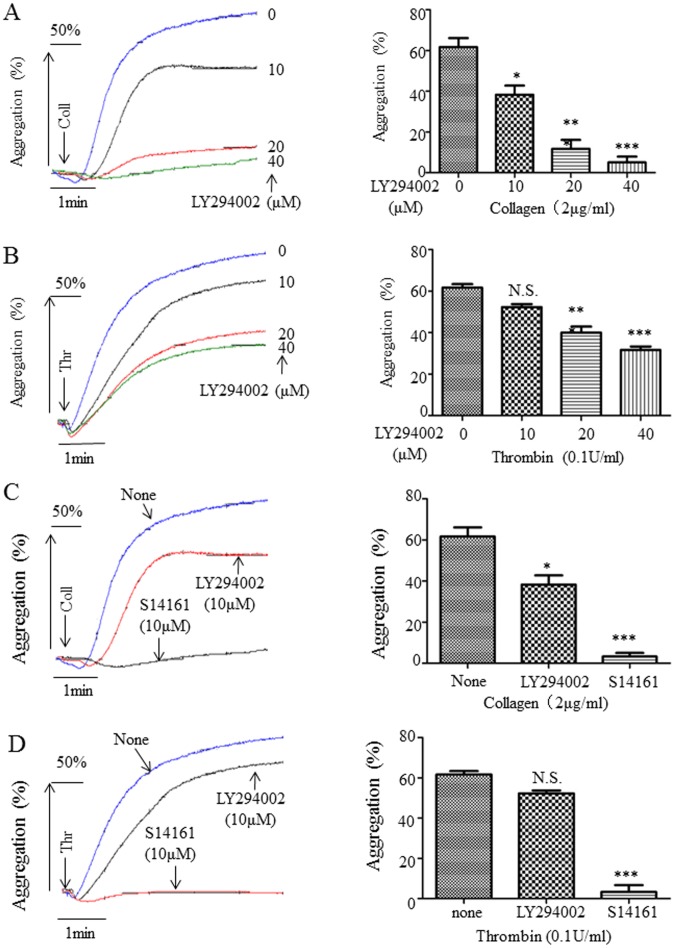
S14161 inhibited platelet aggregation more potently than LY249002. Gel-filtered human platelets (2.5×10^8^/mL) were preincubated for 10 minutes with different concentrations of LY249002 (10 µM, 20 µM and 40 µM) (A, B) or equal concentration of S14161 or LY249002. Platelet aggregation was initiated with collagen (2 µg/mL), thrombin (0.1 U/mL). The aggregation curves are the representatives of at least three individual experiments. Means ± standard errors of the mean of platelet aggregation percentage from three experiments are plotted in the bar charts; *P<0.05, **P<0.01 and ***P<0.001 as compared with control. N.S. means no significance (n = 3).

To compare the inhibition potency of S14161 with LY249002 in platelet aggregation, we also examined LY249002 effect on inhibiting human platelet aggregation induced by collagen (Fig. 2A), thrombin (Fig. 2B), and U46619 (data not shown). To our surprise, LY249002 showed weaker inhibition as compared with S14161, and the IC50s of LY249002 in inhibiting platelet aggregation were much higher than S14161, which are 14.19±1.38 (µM) for collagen (2 µg/ml), 35.12±4.21 (µM) for thrombin (0.1 U/ml), and 25.12±3.72 (µM) for U46619 (1 µM). To further confirm this, we compared the ability of S14161 and LY249002 in inhibiting platelet aggregation using the same concentrations (10 µM) for both inhibitors. Results showed that S14161 almost completely blocked collagen- and thrombin-induced platelet aggregation. However, LY249002 only weakly inhibited platelet aggregation induced by collagen, and has no significant inhibition on thrombin-induced platelet aggregation at this concentration (Fig. 2C &2D). A high concentration of LY249002 (25 µM) showed a comparable inhibition on platelet aggregation as S14161 (10 µM) (data not shown).

**Figure 3:**
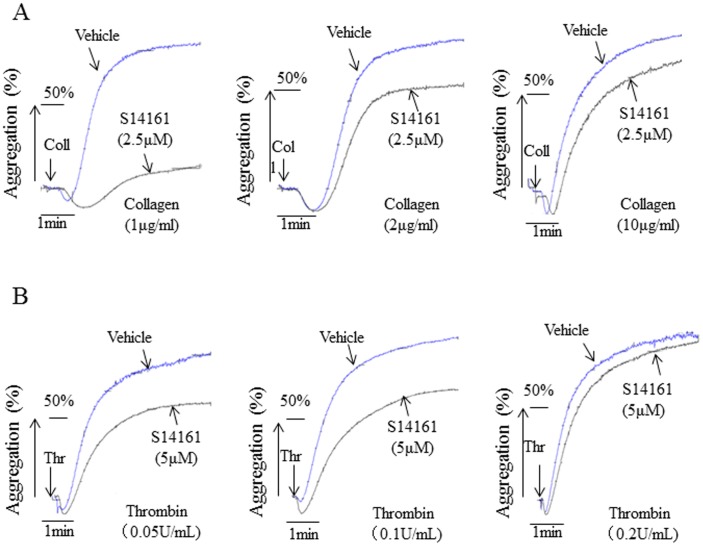
S14161 was unable to disrupt platelet aggregation induced by higher doses of agonists. Gel-filtered platelets (2.5×10^8^/ml) were pre-incubated for 10 min with S14161 (2.5 µM or 5 µM). Platelet aggregation was initiated with different concentration of collagen (1 µg/ml, 2 µg/ml, and 10 µg/ml) (A) or with thrombin (0.05 U/ml, 0.1 U/ml, and 0.2 U/ml) (B). The data are representatives of at least three individual experiments.

In addition, we examined whether S14161 was effective in disrupting platelet aggregation induced by high doses of collagen or thrombin. We preincubated platelets with the minimal concentrations of S14161 (2.5 µM) that significantly inhibits platelet aggregation induced by collagen based on Figure 1A. Different concentrations of collagen (1, 2, or 10 µg/mL) were used to induce platelet aggregation. Results showed that 2.5 µM of S14161 did not inhibited platelet aggregation when collagen concentration reached to 10 µg/ml (Fig. 3A). Similarly, 5 µM of S14161 was found to loss its ability to inhibit platelet aggregation when thrombin concentrations reached to 0.2 U/ml (Fig. 3B). Therefore, S14161 was unable to disrupt platelet aggregation induced by higher doses of agonists.

### S14161 does not induce toxic or apoptotic effects on platelets

**Figure 4:**
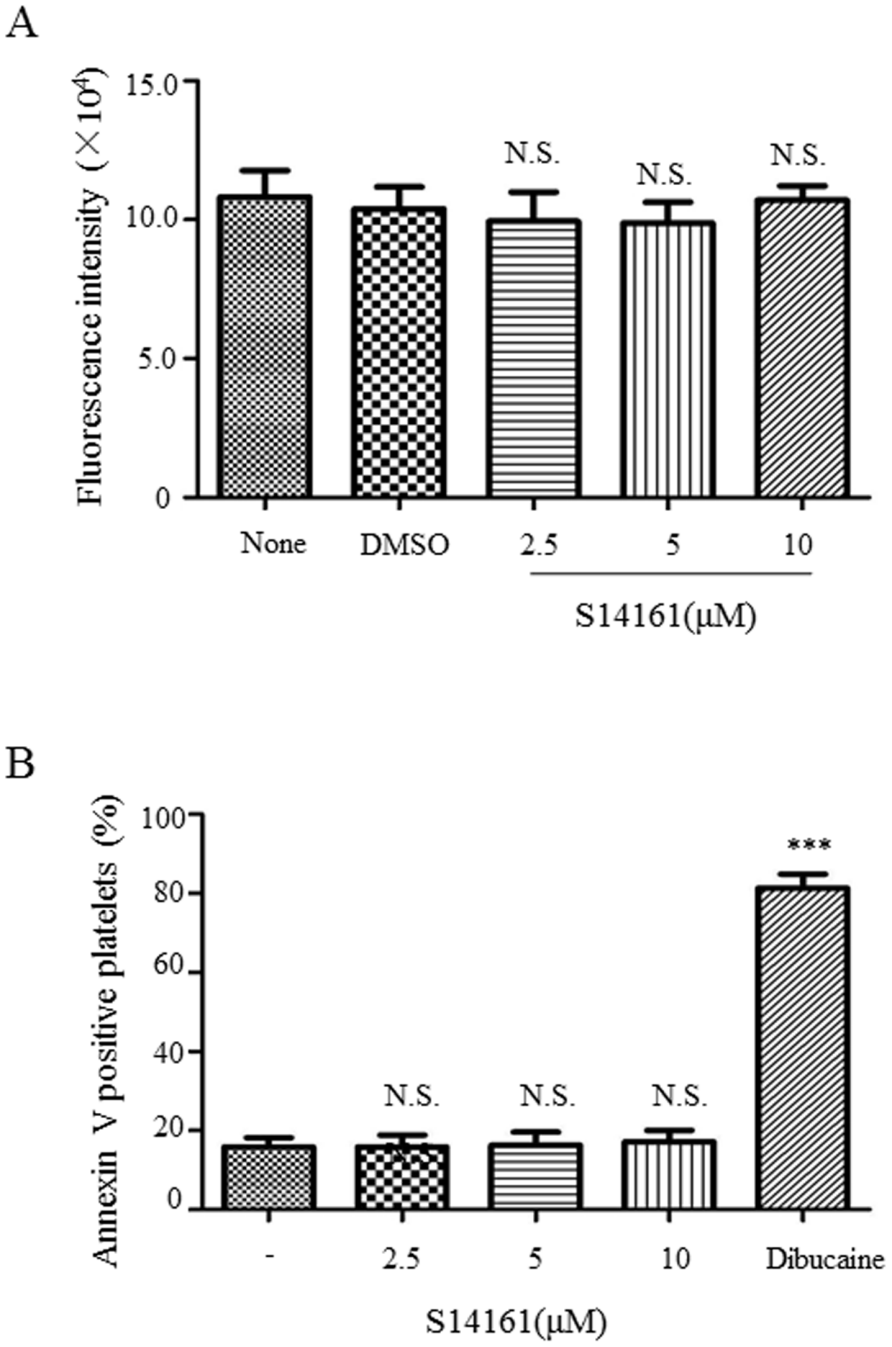
Evaluation of the toxic and apoptotic effect of S14161 on platelets. (A) Gel-filtered platelets (2.5×10^8^/ml) were preincubated with S14161 (2.5 µM, 5 µM and 10 µM) or vehicle and then incubated with Alamar Blue reagent for 4 hours at 37°C. The fluorescence was acquired with the excitation and emission wavelengths of 570 nm and 585 nm, respectively, and the Tyrode buffer control-subtracted fluorescence was plotted in the bar charts (mean ± standard error, n = 3). (B) Gel-filtered platelets (2×10^7^/ml) were preincubated with S14161 (2.5, 5, and 10 µM), vehicle, or dibucaine (500 µmol/L) at 37°C for 15 min, and then mixed with Annexin V binding buffer and Annexin V-APC. Samples were analyzed by flow cytometry and the percentage of annexin V positive platelets was calculated (mean ± standard error, n = 3). ***P<0.001. N.S., not significant compared to vehicle control.

To examine whether the inhibitory effect of S14161 on agonist-induced platelet aggregation is due to its potential toxicity to platelets, we performed an AlamarBlue assay which is based on the reducing power of living platelets that reduces the resazurin, a major component of AlamarBlue, to resorufin, a compound that is highly flourescent. Platelets were exposed to S14161 for 15 minutes at 37°C and then incubated with AlamarBlue cell viability reagent for 4 hours at 37°C. Results showed that no decreases in the fluorescence intensity in the platelet samples treated with S14161 at 2.5, 5, or 10 µM compared to the vehicle control, indicating that treatment of platelets with S14161 in our experimental system does not cause significant toxicity to platelets (Fig. 4A).

To evaluate whether the inhibition of platelet aggregation by S14161 is due to platelet apoptosis because S14161 has been shown to induce myeloma and leukemia cell apoptosis [23], we performed an Annexin V staining assay in which Annexin V specifies phosphatidylserine (PS) exposure on the outside of plasma membrane. The results showed that the percentage of Annexin V positive platelets was not significantly changed by S14161 (Fig. 4B). In contrast, as a positive control, calpain activator dibucaine induced platelet apoptosis [31]. Thus, it was confirmed that the inhibitory effect of S14161 on platelet aggregation does not result from its apoptotic effect.

### S14161 inhibits PI3K/AKT/GSK3β signaling in platelets

**Figure 5:**
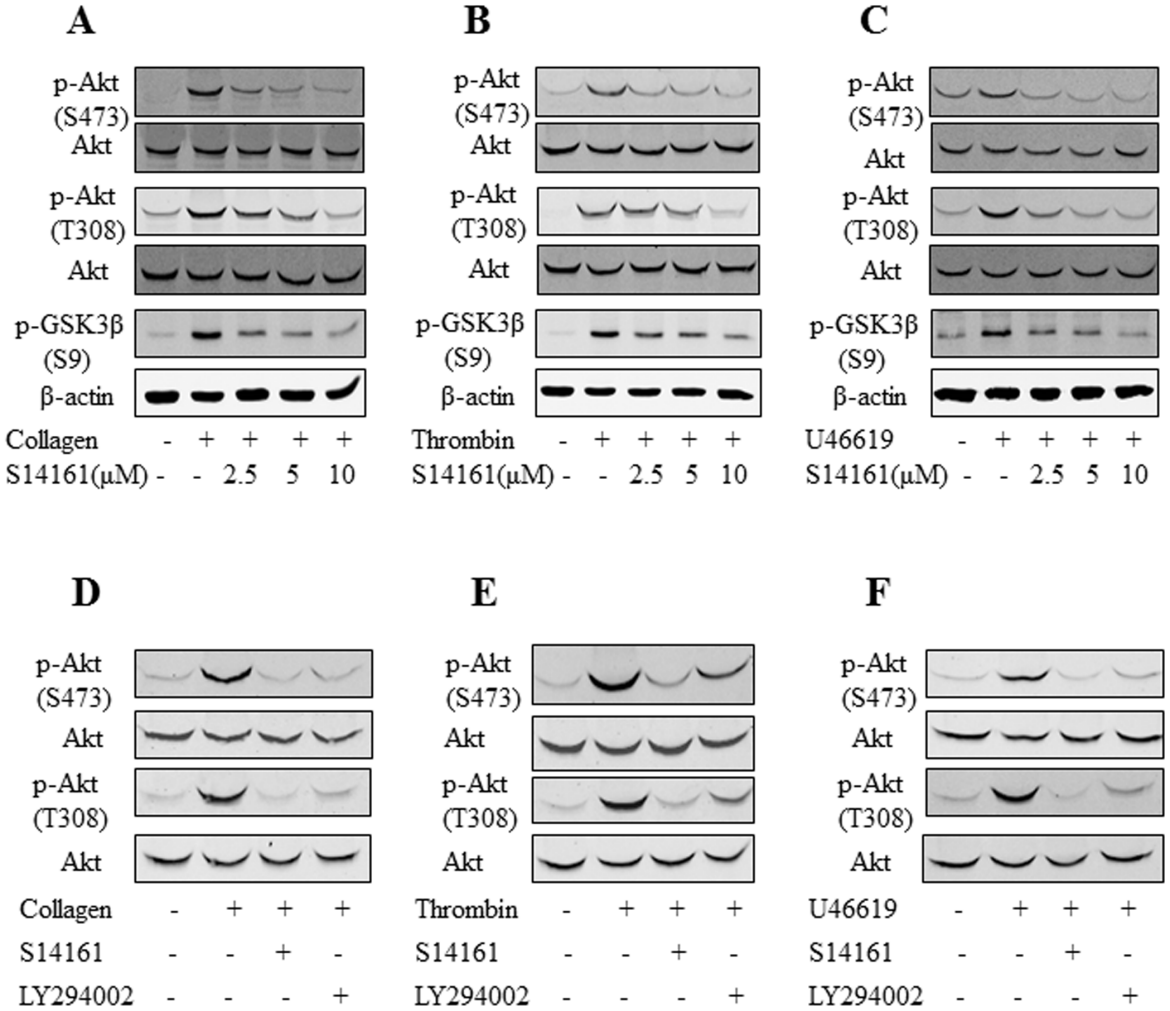
S14161 suppresses agonist-stimulated platelet PI3K signaling phosphorylation. Gel-filtered human platelets (2.5×10^8^/mL) were preincubated with or without S14161 (2.5 µM, 5 µM and 10 µM) or LY294002 (10 µM). Then, platelets were stimulated with collagen (2 µg/mL), thrombin (0.1 U/mL) or U46619 (1 µM) for 3 min with stirring at 1000 r.p.m. in an aggregometer at 37°C. Platelets were lysed, and immunoblotted using the corresponding antibodies recognizing total or phosphorylated AKT (Ser473 or Thr308) and phosphorylated GSK3β (Ser9). Densitometric band scanning was performed using an Odyssey Infrared Imaging System (LI-COR Biosciences). Data are representatives of at least 3 independent experiments.

PI3K is a critical transmitter of intracellular signaling during platelet activation. Because the PI3K pathway is the most susceptible signaling pathway to S14161 [23], we further investigated how S14161 inhibits PI3K signaling in platelets. Gel-filtered human platelets were preincubated with or without S14161 (37°C, 10 minutes) before being challenged by collagen, thrombin, or U46619, and the phosphorylation of AKT, an indicator of PI3K pathway activation was examined. Results showed that phosphorylation of AKT at both Ser473 and Thr308 sites induced by collagen, thrombin, or U46619 were significantly inhibited by S14161 in a dose-dependent manner (upper and middle panels in Fig. 5A–5C). As AKT mediates phosphorylation and inhibition of glycogen synthase kinase-3β (GSK3β), which is a negative regulator of platelet function [32], we examined the effect of S14161 on the phosphorylation of GSK on Ser9 in platelets. Results showed that S14161 attenuated platelet GSK phosphorylation in response to collagen, thrombin, and U46619 (lower panels in Fig. 5A–5C). Therefore, these data indicated that S14161 inhibits platelet activation via the PI3K/AKT/GSK3β pathway.

As S14161 showed a more potent inhibition in platelet aggregation, we compare its potency in inhibition of AKT phosphorylation with LY294002 as well. The same dose of the two compounds (10 µM) was used to pretreat platelets that were challenged by collagen, thrombin, and U46619. However, S14161 showed a comparable potency with LY294002 in inhibiting AKT phosphorylation (Fig. 5D–5F).

### Influence of S14161 on platelet alpha-granule secretion and integrin inside-out signaling

**Figure 6:**
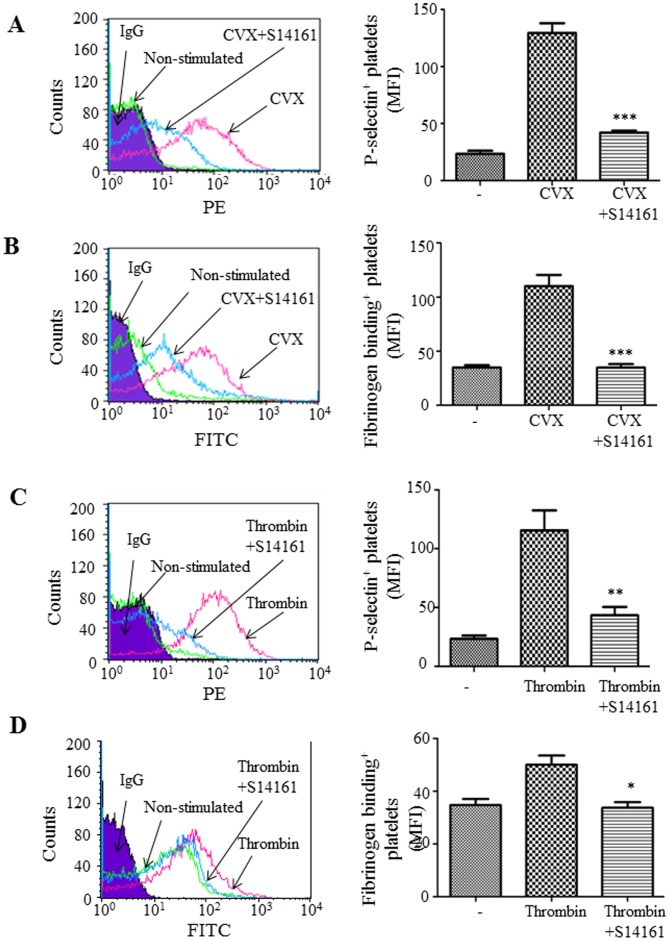
Influence of S14161 on platelet alpha-granule secretion and integrin inside-out signaling. Gel-filtered human platelets (2×10^7^/mL) were preincubated with S14161 (10 µM) at 37°C for 10 minutes in the presence of fluorescent-conjugated antibodies against P-selectin or fibrinogen. Samples were then challenged with convulxin (0.5 nM) or thrombin (0.1 U/mL), and incubated for an additional 15 minutes. P-selectin expression (A, C) and fibrinogen binding (B, D) of single platelet were monitored by Flow cytometry. Data plotted are means ± standard errors of the mean; n = 3; *P<0.05, **P<0.01 and ***P<0.001 with vs. without S14161 treatment.

To examine the effects of S14161 on platelet secretion, human platelets were pre-incubated with S14161 (10 µM) in the presence of fluorescent PE-conjugated P-selectin antibody and then challenged with convulxin (0.5 nM) for 15 minutes. P-selectin expression on platelets was monitored by flow cytometry. Results showed that S14161 inhibited convulxin- induced P-selectin expression by 67.5% (P<0.001) (Fig. 6A). To investigate whether S14161 affected integrin inside-out signaling, we examined the effects of S14161 on integrin αIIbβ3 activation using soluble fibrinogen, which can bind to activated integrin αIIbβ3. Results showed that S14161 inhibited convulxin-induced platelet integrin activation by 68.3% (P<0.001) (Fig. 6B).

To examine the inhibitory effect of S14161 on platelet alpha-granule secretion and integrin inside-out signaling induced by other common agonists such as thrombin, we performed p-selectin expression and fibrinogen binding assays using thrombin (0.1 U/ml) as an agonist. We showed that S14161 inhibited thrombin-induced platelet alpha-granule secretion and integrin inside-out signaling (Fig. 6C &. 6D). Additional experiments showed that lower doses of S14161 (2.5 and 5 µM) also showed significant inhibition on convulxin-induced P-selectin expression and fibrinogen binding to a lesser extent. Although the same doses of S14161 showed significant inhibition for convulxin-induced fibrinogen binding, this inhibition did not occur for thrombin-induced fibrinogen binding (data not shown). Taken together, these data suggested that platelet secretion and integrin inside-out signaling were modulated by S14161.

### Effects of S14161 on integrin outside-in signaling in platelets

**Figure 7:**
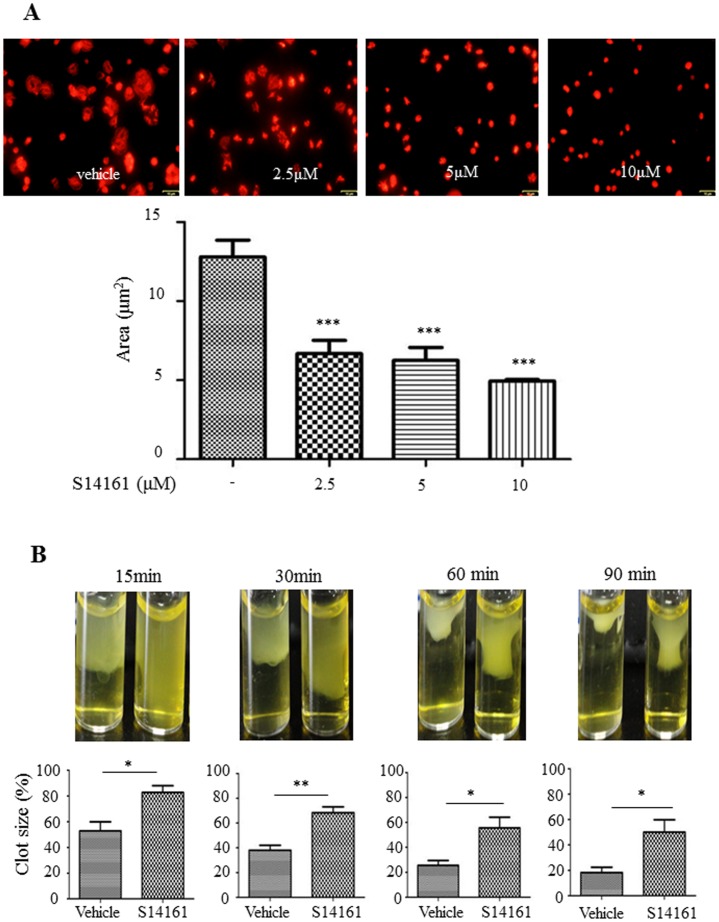
Effects of S14161 on platelet spreading on immobilized fibrinogen and fibrin clot retraction. (A) Gel filtered human platelets (2×10^7^/mL) were preincubated with S14161 (2.5 µM, 5 µM and 10 µM) or vehicle for 30 min at 37°C and allowed to spread on fibrinogen-coated slides. Images were obtained with an Olympus fluorescence microscope. Statistical data were derived from quantitative results (means ± SEM) calculated from the mean of the average surface area of individual platelets from 3 separate experiments. ***P<0.001 vs. vehicle control. (B) Platelet rich plasma was incubated with S14161 (10 µM) or vehicle. Fibrinogen (2 mg/mL) was added and fibrin clot formation was initiated by adding 1 U/mL of thrombin. Clot retraction was monitored over time, and photographs of the clots were taken at different time points. The histograms of the clot size were generated from the photographs by calculating the ratio of the surface area of the retracted clot versus that of the initial clot. *P<0.05 and **P<0.01 compared with vehicle control (n = 3).

As S14161 showed potent inhibition of platelet aggregation and fibrinogen binding, reflecting platelet inside-out signaling, we further asked whether S14161 could affect integrin outside-in signaling. Platelet spreading on immobilized fibrinogen assay was performed. As shown in figure 7A, the average surface coverage of vehicle-treated platelets was 12.79±2.91 µm^2^, which was significantly reduced by S14161 in a dose dependent manner. At 10 µM, S14161 reduced platelet surface coverage by 61.3% (4.95±0.27 µm^2^) as compared with the vehicle control. Platelet clot retraction is an essential step in platelet thrombus consolidation and is initiated after platelet activation as a late consequence of integrin outside-in signaling. It also relies on an efficient extracellular fibrinogen-integrin interaction as well as a stable integrin-cytoskeletal association [28]. To investigate whether S14161 interferes with this important platelet function, we performed platelet clot retraction assay using PRP pretreated with S14161 (10 µM) or vehicle. Fibrin clots were induced at 37°C by the addition of thrombin to PRP and the subsequent platelet-dependent clot retraction was monitored over a 90-minute time period. As shown in figure 7B, the fibrin clots in the vehicle control tubes underwent a time-dependent retraction that started at 15 minutes and was completed at 90 minutes. The presence of S14161 at 10 µM resulted in a significant delay of clot retraction.

### Inhibition of platelet adhesion on collagen-coated surfaces under flow by S14161

**Figure 8:**
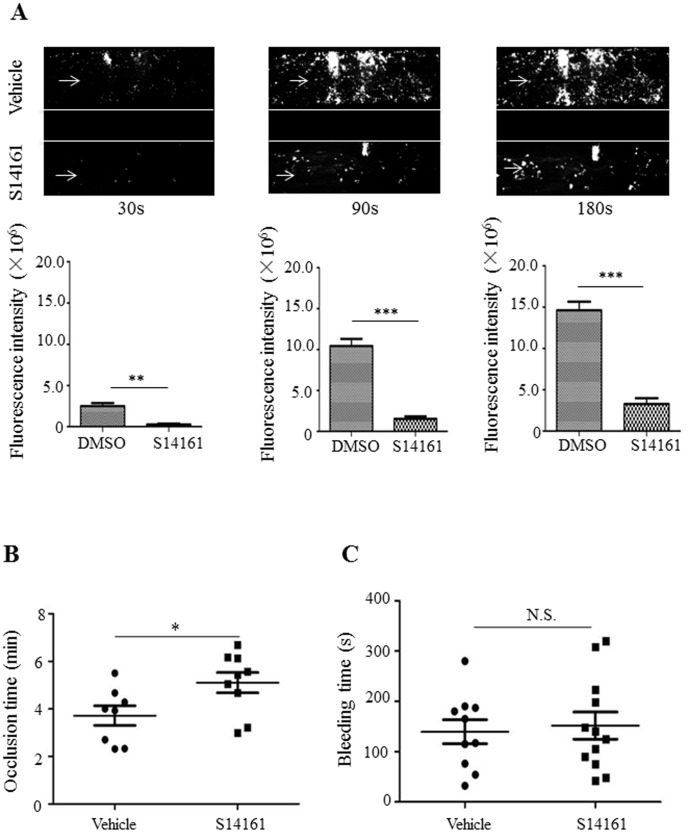
S14161 inhibited platelet adhesion on collagen-coated surfaces under flow ex vivo and thrombus formation in vivo, but not hemostasis. (A) Human whole blood was labeled with Calcein-AM, treated with (lower channels) or without (upper channels) S14161 (10 µM), and perfused over collagen-coated surface at a shear rate of 40 dyn/cm^2^ (1000 s^−1^). Platelet adhesion and aggregates were photographed. Data plotted are means ± standard errors of fluorescence intensity of three individual experiments (**P<0.01, ***P<0.001). Arrow indicates the flow direction. (B) S14161 (2 mg/kg) (n = 9) or vehicle (n = 8) was administered intraperitoneally to mice and time to occlusion of the carotid artery after the application of 7.5% FeCl_3_ for two minutes was measured using Visual Sonics View 2100. Time to loss of blood flow was recorded. (C) S14161 (2 mg/kg) (n = 12) or vehicle (n = 10) was administered intraperitoneally to mice. The bleeding end was immersed in saline at 37°C, and the time to bleeding cessation was recorded. *P<0.05 vs. vehicle control. N.S. means no significance.

To further examine the inhibitory effect of S14161 on platelet adhesion on a collagen-coated surface, we perfused anticoagulated blood that had been labeled by calcein-AM and preincubated with S14161 over a collagen-coated surface in the microfluidics flow chamber (1000 s^−1^). The fluorescence intensity from platelets on the collagen surface was calculated and data analysis showed that at a shear rate of 1000 s^−1^, S14161 (10 µM) inhibits platelet accumulation by 77.6% (P<0.01), 85.4% (P<0.001), and 88.9% (P<0.001) at 30 s, 90 s, and 180 s, respectively (Fig. 8A).

### S14161 significantly affects thrombus formation, but not hemostasis, *in vivo*

To make sure that S14161 could be used for the *in vivo* animal studies on its potential inhibitory effect on thrombus formation as well as for the future clinical study, we performed a series of platelet aggregations using PRP, showing that S14161 significantly inhibited platelet aggregation induced by collagen (2 µg/ml), and U46619 (1 µmol/L) in human and mouse PRP (data not shown). To test whether S14161 inhibits arterial thrombus formation in vivo, S14161 (2 mg/kg) (n = 9) or vehicle (n = 8) was administered intraperitoneally to mice (6–8 weeks) and the time to occlusion of the carotid artery after application of 7.5% FeCl_3_ for 2 minutes was measured using VisualSonics View 2100. Results showed that S14161-treated mice significantly extended the first occlusion time (5.05±0.99 min, N = 9) when compared to the vehicle-injected mice (3.72±0.95 min, n = 8) (P<0.05) (Fig. 8B). To examine whether S14161 affects hemostasis while inhibiting platelet activation and thrombus formation, tail-bleeding time was evaluated. Results showed that S14161 did not significantly prolong the tail bleeding time (151.8±73.6 sec; n = 12) in mice that received a dose of 2 mg/kg S14161 compared to the vehicle-treated mice (139.6±71.3 sec; n = 10) (Fig. 8C). Therefore, S14161 appears to inhibit thrombus formation, but not cause a prolonged bleeding time.

## Discussion

In this study we showed that S14161 inhibits platelet aggregation in the presence of a variety of platelet agonists including collagen, thrombin, ADP and U46619. S14161 also inhibits platelet secretion, integrin activation, and platelet adhesion on a collagen surface. Mechanistically, S14161 inhibits platelet function by suppressing PI3K/AKT signaling pathway. *In vivo* studies showed that S14161 prolongs the occlusion time upon vascular injury without bleeding tendency.

We previously showed that S14161 is a novel chromene-based inhibitor of pan-class I PI3Ks that inhibits the production of PI(3,4,5)P3 thus suppressing PI3K/AKT signaling transduction and displaying preclinical activity efficacy against multiple myeloma and leukemia without overt toxicity *in vitro* and *in vivo*, [23]. The cell free enzymatic assay showed that S14161 inhibited PI3K isoforms with an IC50 of around 65 to 110 µM [23], which are higher than LY294002 (IC50: 1.4 µM) [33], the first synthetic prototypic inhibitor of pan-PI3K. However, our multiple sets of experiments showed that its potency in inhibiting platelet aggregation is higher than LY294002. To fully understand this discrepancy, we compared the IC50s of S14161 with LY294002 in inhibiting the ability of PI3K isoforms to generate PI(3,4,5)P3 in a cell free enzymatic assay [23]. Our data showed that IC50 of S14161 (PI3Kα: 45.22; PI3Kβ: 138.0; PI3Kγ: 85.46; PI3Kδ: 121.0) was up to 89 fold higher than LY294002 (PI3Kα: 0.64; PI3Kβ: 1.55; PI3Kγ: 3.43; PI3Kδ: 2.74). The difference of the potency between S14161 and LY294002 in inhibiting the *in vitro* enzymatic activity and in inhibiting platelet aggregation may be because S1 has a better bioavailability in the natural system, such as in the cells or in the mice.

PI3Ks form the lipid second messenger PI(3,4,5)P3 at the plasma membrane upon platelet activation and recruit phosphoinositide-dependent protein kinase 1 (PDK1) and AKT. PDK1 phosphorylates AKT on Thr308 and AKT is subsequently phosphorylated on Ser473 by mTORC2, a key downstream mediator of PI3K-dependent signaling, resulting in maximal AKT activity [34], [35]. In our study, S14161 decreases phosphorylation of AKT at both Thr308 and Ser473 sites in a similar potency in response to collagen, thrombin, and U46619. Inhibition of S14161 on PI3K/AKT was further confirmed by the downregulated phosphorylation of GSK-3β (Ser9). GSK-3 tonically active in resting cells but inhibited by phosphorylation of an N-terminal Ser21 in GSK3α and Ser9 in GSK3β in response to varied external stimuli [36]. As GSK-3β is a major target of the PI3K/AKT signaling pathway, we examined effect of S14161 on agonist-induced phosphorylation of GSK-3β (Ser9), showing that S14161 inhibited GSK-3β phosphorylation.

Platelet receptors receive stimulations from adhesive proteins exposed in an injured vessel wall and soluble platelet agonists, transmit the signals via their platelet activation pathway, and ultimately induce the “inside-out” signaling process that leads to integrin activation [37]. PI3K/AKT signaling is one of the important pathways to mediate receptor signals, activating CalDAGGEF1, Rap1B and RIAM pathway to promote αIIbβ3-talin interaction and integrin activation. Apparently, S14161 inhibited platelet inside-out signaling as it attenuates fibrinogen binding by the selective collagen receptor ligand convulxin and thrombin. Although the detail mechanism is unclear, inhibition of PI3K/AKT/GSK-3β signaling by S14161 should be the major event to modulate inside-out signaling. Integrin signaling is bidirectional that intracellular signaling induces changes in the extracellular ligand–binding domain of integrins to an activated state. Ligand binding to the activated integrins conversely transmits outside-in signals, which are critically important in stable platelet adhesion, spreading, and clot retraction as reviewed by Li Z, et al [37]. We chose platelet spreading, an early consequence of integrin outside-in signaling characterized by the formation of lamellipodia and filipodia, and clot retraction, a process driven by integrin outside-in signaling of platelet integrin αIIbβ3, to evaluate whether S14161 inhibits this process. As expected, S14161 inhibited not only platelet inside-out signaling but also integrin outside-in signaling, processes known to be strongly regulated by PI3K.

The effect of S14161 was further evaluated by *ex vivo* and *in vivo* assays. S14161-treated platelets showed less adhesion and accumulation than the untreated platelets at 1000 s^−1^, which may be due to decreased platelet activation. Since GPIb complex-VWF interaction is important for platelet adhesion at high shear stress, the effect of S14161 on platelet adhesion at a higher rate will be tested in our future studies [38], [39]. For *in vivo* studies, we examined the effect of S14161 on thrombus formation using FeCl3-induced carotid artery injury model, which measures the thrombosis in the medium or large artery, and showed that S14161 negatively regulates thrombus formation. The benefit of an antithrombotic agent has to be considered under the balance between efficacy in reduction of symptomatic thrombotic events and the risk for hemorrhage. Our study confirmed that S14161 does not cause a prolonged bleeding time.

It is reasonable to suspect that S14161 may have side effects as other PI3K inhibitors do when they are used as a therapeutic agent. However, our previous studies [23] and the data on platelets in the present study have shown that S14161 is a safe compound in the safety evaluation. S14161 delayed the growth of leukemia xenografts in nude mice within 10 days but did not shown any obvious toxicity. Although S14161 induces apoptosis in myeloma and leukemia cell lines and primary patient samples, it does not induce apoptosis in platelets. The other potential issue needs to be clear is the specificity of S14161 in inhibiting platelet activation especially as it shows discrepancy in inhibiting platelet aggregation and PI(3,4,5)P3 generation *in vitro*. However, in our previous work [23], we showed that it did not inhibit the enzymatic activities of other related kinases, including the mammalian target of rapamycin, the DNA-dependent protein kinase catalytic subunit, and phosphoinositide-dependent kinase-1. Further investigation need to be done for the specificity of S14161.

In summary, this study demonstrated for the first time the inhibitory effect of S14161 on platelet activation and thrombus formation without significant bleeding tendency. Comparing to LY249002, S14161 displayed a more potent effect on inhibiting platelet aggregation potentially due to its better bioavailability in the natural system. Further study in comparing its antithrombotic effect and bleeding tendency with other well-studied PI3K inhibitors would generate more information on its advantages in effectiveness and safety as it mainly affects platelet PI3K/AKT signaling. Collectively, our data in this study and our previous reports clearly suggest that this chemical compound may be developed as a potential antiplatelet and antithrombotic agent for the prevention and treatment of thrombotic disease.
